# Perceived Credibility of Social Media Narratives About GLP-1 Receptor Agonists and Acceptance of Integrated Obesity Care: Implications for Nutrition and Lifestyle Support

**DOI:** 10.3390/nu18162718

**Published:** 2026-08-20

**Authors:** Tomasz Żurawski, Anna Bartosiewicz

**Affiliations:** 1 University of Rzeszów, Collegium Medicum, Faculty of Health Sciences and Psychology, 35-959 Rzeszów, Poland; tz088246@stud.ur.edu.pl; 2 University Center for Research and Development in Health Sciences, 35-310 Rzeszów, Poland

**Keywords:** GLP-1 receptor agonists, obesity pharmacotherapy, social media, perceived credibility, health misinformation, integrated obesity care, nutrition and lifestyle care

## Abstract

**Background/Objectives:** GLP-1 receptor agonists are an established component of obesity treatment that should complement nutritional and lifestyle interventions. Social media narratives may shape public understanding and acceptance of these therapies. This study examined whether exposure to GLP-1-related narratives and their perceived credibility were associated with perceptions of obesity pharmacotherapy. **Methods:** An anonymous cross-sectional online survey was conducted among Polish-speaking adults aged ≥ 18 years who used social media at least weekly and reported exposure to GLP-1-related content within the preceding 3 months. Five exploratory composite scores assessed narrative exposure, perceived credibility, perceptions of communication, attitudes toward obesity pharmacotherapy, and GLP-1 treatment evaluation. Associations were examined using univariable and multivariable linear regression. **Results:** The final sample comprised 279 participants (84.6% women; mean age, 38.14 ± 8.58 years). In adjusted models, greater perceived narrative credibility was associated with less favorable GLP-1 treatment evaluations (*B* = −0.311, 95% CI: −0.359 to −0.263; *p* < 0.001) and greater endorsement of negative pharmacotherapy-related statements (*B* = 0.401, 95% CI: 0.350 to 0.453; *p* < 0.001). Greater narrative exposure was associated with more favorable GLP-1 treatment evaluations (*B* = 0.072, 95% CI: 0.026 to 0.118; *p* = 0.002) and more negative perceptions of social media communication (*B* = 0.235, 95% CI: 0.161 to 0.309; *p* < 0.001). **Conclusions:** Greater perceived credibility of GLP-1-related narratives was associated with less favorable perceptions of obesity pharmacotherapy, but the cross-sectional design precludes conclusions about direction or causality. The findings highlight the importance of evidence-based communication that supports critical evaluation of treatment-related claims and presents pharmacotherapy, nutrition, and lifestyle support as complementary components of comprehensive obesity care.

## 1. Introduction

Obesity represents a major global public health challenge. Recent global estimates indicate that approximately 890 million adults were living with obesity in 2022, and its global prevalence has more than doubled since 1990 [1]. Obesity is associated with an increased risk of numerous chronic conditions, reduced quality of life, and a substantial burden on healthcare systems [2].

Contemporary approaches to obesity increasingly recognize it not merely as a problem of excess body weight, but as a complex, chronic disease characterized by excessive adiposity and its adverse effects on organ function and overall health [2]. In response to the growing burden of obesity, a range of therapeutic strategies has been developed, including nutritional and lifestyle interventions [3], pharmacotherapy [4], and bariatric surgery [5].

GLP-1 receptor agonists have attracted particular attention because they have been shown to produce clinically meaningful weight loss, with clinical relevance linked to their effects on appetite regulation, satiety, gastric emptying, glucose homeostasis, and cardiometabolic risk factors [6,7]. Consequently, these medications now play an important role in contemporary obesity treatment and are widely discussed both within the medical community and beyond it. Across chronic conditions, patients’ beliefs about the necessity of prescribed medicines and concerns about potential adverse effects are consistently associated with medication adherence. These beliefs by shaping adherence may affect the benefits achieved in routine care [8]. In obesity care, although the available evidence remains limited, treatment with GLP-1 receptor agonists may be accompanied by stigmatizing perceptions, including its characterization as a shortcut and moral judgments about personal responsibility for weight management. Such framing may be relevant to whether pharmacotherapy is regarded as a legitimate component of obesity treatment [9]. At the same time, people seeking information about health and treatment are increasingly turning to online sources, including social media [10,11].

Pharmacotherapy is one component of comprehensive obesity treatment, alongside nutritional interventions, physical activity, and broader behavioral support [4]. Current recommendations emphasize that GLP-1 receptor agonist therapy should be embedded within a model of care that includes appropriate nutritional and lifestyle support rather than being presented as a substitute for these interventions [12,13]. The way in which GLP-1 receptor agonists are portrayed may therefore shape not only perceptions of pharmacotherapy itself, but also how the relationship between medication, nutritional therapy, and behavioral interventions is understood. This is particularly relevant in social media environments, where simplified messages may portray medication use and engagement in dietary and lifestyle change as opposing approaches to obesity treatment [9,14,15]. As social media plays an increasingly prominent role as a source of health information, greater attention is being directed toward the quality of the content disseminated through these platforms. Health misinformation has been recognized as an important public health challenge, and available reviews indicate that the quality of health and nutrition information published online remains highly variable [14,15].

Although existing studies have examined the quality of health information shared on social media and the prevalence of health misinformation, considerably less is known about how exposure to such content and the credibility attributed to it are associated with perceptions of specific treatment modalities. Evidence remains particularly limited regarding obesity pharmacotherapy and GLP-1 receptor agonists. It is also unclear whether associations between such messages and treatment perceptions are primarily related to exposure itself or to the credibility attributed to the narratives they convey. Distinguishing between exposure to content and its perceived credibility may provide a more detailed understanding of the factors associated with perceptions of obesity pharmacotherapy in the social media environment. Clarifying these relationships may inform the development of educational interventions and communication strategies concerning obesity treatment.

The aim of this exploratory cross-sectional study was to examine whether exposure to social media narratives concerning GLP-1 receptor agonists and the perceived credibility of these narratives were associated with perceptions of obesity pharmacotherapy. Specifically, we assessed their associations with evaluations of GLP-1-based obesity treatment, attitudes toward obesity pharmacotherapy, and perceptions of social media communication about obesity treatment. By distinguishing exposure from perceived credibility, we examined whether the appraisal of GLP-1-related narratives was associated with evaluations and attitudes relevant to the acceptance of pharmacological treatment for obesity.

## 2. Materials and Methods

### 2.1. Study Design and Participant Recruitment

An anonymous, cross-sectional exploratory online study was conducted using an original web-based questionnaire developed in Google Forms (Google LLC, Mountain View, CA, USA). Data collection was conducted between late May and late June 2026. Participants completed the questionnaire independently in an online setting.

A non-probability convenience sampling strategy with elements of snowball sampling was used. Recruitment was conducted through several social media channels. The survey link was disseminated through publicly accessible social media accounts managed by the authors and posted in thematic groups related to health, nutrition, physical activity, and weight loss. It was subsequently shared further by group administrators and other social media users within their own networks. No paid promotion or other forms of paid survey distribution were used. Participation was voluntary and involved no financial compensation or material incentives.

**Figure 1:**
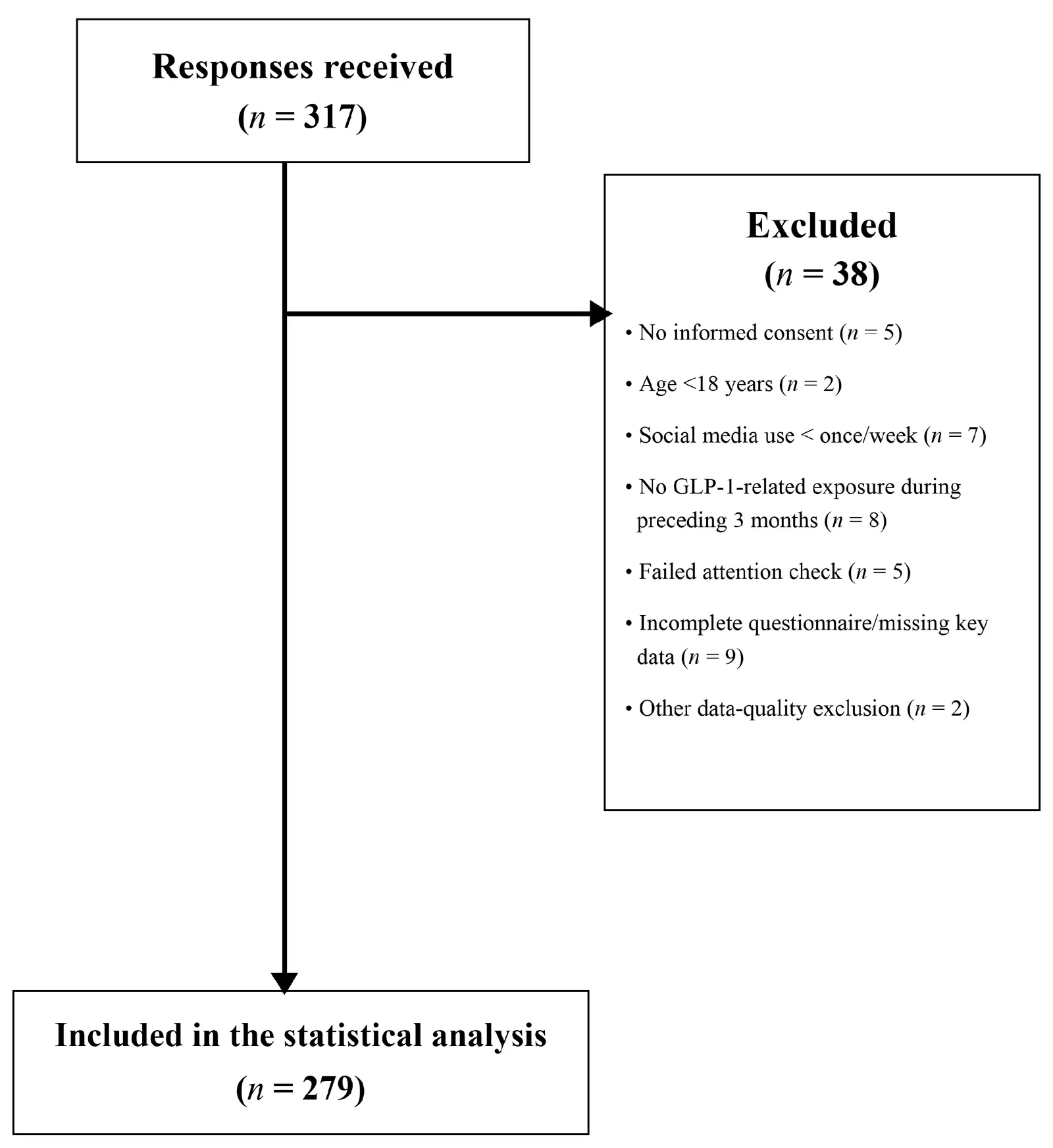
Flow chart of participant recruitment, eligibility assessment, and reasons for exclusion.

Because the survey link was distributed publicly and could be further shared by social media users, it was not possible to calculate a conventional response rate. The participant selection process is presented in Figure 1.

### 2.2. Eligibility and Exclusion Criteria

Eligible participants were adults aged ≥18 years who reported proficiency in Polish and used social media at least once per week. Only participants who reported prior exposure to social media content related to GLP-1 receptor agonists within the preceding 3 months were included in the analyses.

Individuals were excluded if they did not provide informed consent, were younger than 18 years, used social media less than once per week, had not encountered GLP-1-related social media content during the preceding 3 months, failed the attention-check item, submitted an incomplete questionnaire or had missing key data or met other prespecified data-quality exclusion criteria related to response consistency or plausibility. The number of responses excluded for each reason is presented in Figure 1.

The questionnaire items concerning height and body weight were optional. Missing values for either variable did not constitute grounds for exclusion because these data were used solely to calculate BMI, which was treated as a covariate rather than an eligibility criterion or study outcome. All participants included in the final sample had complete data required to calculate the principal composite scores. BMI could be calculated for 268 of the 279 participants. Missing values were not imputed, and models including BMI were conducted using complete cases.

### 2.3. Questionnaire and Data Collection

The questionnaire was developed specifically for this study and comprised multiple sections. It included single-choice and multiple-choice questions, demographic items, and sets of statements rated using five-point response scales.

The first section of the questionnaire provided information about the study and included the informed consent form. This was followed by screening questions concerning age, social media use, and prior exposure to social media content related to GLP-1 receptor agonists within the preceding 3 months.

Subsequent sections assessed the frequency of exposure to GLP-1-related social media content; the platforms, formats, and forms of engagement through which participants encountered such content; exposure to selected narratives concerning GLP-1 receptor agonists; the perceived credibility of those narratives; perceptions of social media communication about obesity treatment; attitudes toward obesity pharmacotherapy; and evaluations of GLP-1-based treatment. The GLP-1-related statements were developed specifically for this study. Their content was informed by recurring messages about obesity pharmacotherapy observed by the authors in Polish-language social media before data collection.

The questionnaire was developed and administered in Polish. The English-language version provided in the Supplementary Materials was prepared to support transparency and reproducibility and was not used during recruitment or data collection. Response anchors for the five-point scales were tailored to the construct assessed.

The final section collected demographic and health-related data, including sex, age, height, body weight, educational attainment, daily time spent using social media, previous use of obesity pharmacotherapy, and self-reported history of an obesity diagnosis. The full questionnaire is provided in English in the Supplementary Materials.

The questionnaire did not undergo formal psychometric validation before data collection. The internal consistency of the composite scores derived from its items was evaluated in the present sample using Cronbach’s alpha and McDonald’s omega total, as described in Section 2.5.

### 2.4. Study Variables and Composite Score Construction

The demographic, health-related, and behavioral variables examined included age, sex assigned at birth, educational attainment, self-reported daily time spent using social media, ever use of GLP-1 receptor agonists for obesity treatment, self-reported history of an obesity diagnosis, and body mass index (BMI). BMI was calculated from self-reported weight and height using the following formula:

BMI (kg/m^2^) = weight (kg)/[height (m)] ^2^

For the regression analyses, BMI was entered as a categorical variable with the following levels: <25.0 kg/m^2^, 25.0 to <30.0 kg/m^2^, 30.0 to <35.0 kg/m^2^, 35.0 to <40.0 kg/m^2^, and ≥40.0 kg/m^2^. BMI < 25.0 kg/m^2^ was used as the reference category.

Ever use of GLP-1 receptor agonists was coded as yes or no. Participants who had never used obesity pharmacotherapy and those who had used only other obesity medications were included in the no-use category. “Prefer not to answer” responses were treated as missing. This variable was used for descriptive purposes only and was not included in the regression models.

The five original categories of daily social media use were retained for descriptive analyses. Because only four participants reported using social media for <30 min/day, this category was combined with 30–60 min/day to form the ≤1 h/day category for the regression analyses. The 1–2 h/day category was retained, whereas 2–4 h/day and >4 h/day were combined into >2 h/day. The ≤1 h/day category was used as the reference.

Five exploratory composite scores were calculated: narrative exposure, perceived narrative credibility, perceptions of social media communication about obesity treatment, attitudes toward obesity pharmacotherapy, and GLP-1 treatment evaluation. Each composite score was calculated as the unweighted sum of its constituent item scores.

The narrative exposure score comprised nine items coded from 0 (“never”) to 4 (“very often”). The possible score ranged from 0 to 36, with higher values indicating more frequent exposure to the narratives under investigation on social media.

The perceived narrative credibility score comprised nine corresponding items coded from 0 (“completely not credible”) to 4 (“completely credible”), with “difficult to assess” coded as 2. The possible score ranged from 0 to 36, with higher values indicating greater credibility attributed to the narratives under investigation.

The perceptions of social media communication score comprised five items coded from 0 (“strongly disagree”) to 4 (“strongly agree”). The possible score ranged from 0 to 20, with higher values indicating stronger agreement with negative appraisals of social media communication about obesity treatment.

The attitudes toward obesity pharmacotherapy score comprised six items coded from 0 (“strongly disagree”) to 4 (“strongly agree”). The positively worded item concerning willingness to consider obesity pharmacotherapy was reverse scored: original values of 0, 1, 2, 3, and 4 were recoded as 4, 3, 2, 1, and 0, respectively. The possible score ranged from 0 to 24. Higher values reflected stronger endorsement of negative pharmacotherapy-related statements and lower willingness to consider such treatment.

The GLP-1 treatment evaluation score comprised four items coded from 0 (“strongly disagree”) to 4 (“strongly agree”). The possible score ranged from 0 to 16, with higher values indicating more favorable evaluations of treatment with GLP-1 receptor agonists.

All five composite scores were analyzed as continuous variables.

### 2.5. Statistical Analysis

Continuous variables were summarized using the mean and standard deviation (SD), median, first and third quartiles (Q1 and Q3), and minimum and maximum values. Categorical variables were summarized as counts and percentages.

The internal consistency of each of the five composite scores was assessed using Cronbach’s alpha and McDonald’s omega total.

Three composite outcomes were analyzed: GLP-1 treatment evaluation, attitudes toward obesity pharmacotherapy, and perceptions of social media communication about obesity treatment. Candidate predictors comprised exposure to narratives concerning GLP-1 receptor agonists, perceived narrative credibility, sex assigned at birth, age, educational attainment, BMI category, and self-reported daily time spent using social media.

Associations between potential predictors and each outcome were examined using univariable and multivariable linear regression models. Predictors with *p* < 0.30 in the univariable analyses were included in the corresponding multivariable model while retaining at least 15 observations per estimated regression parameter. This criterion was applied to limit model complexity relative to the available sample size.

All multivariable models included narrative exposure, age, and educational attainment. Perceived narrative credibility and BMI category were additionally included in the models for GLP-1 treatment evaluation and attitudes toward obesity pharmacotherapy. Daily social media use was included in the models for GLP-1 treatment evaluation and perceptions of social media communication about obesity treatment, whereas sex assigned at birth was included in the models for attitudes toward obesity pharmacotherapy and perceptions of social media communication.

The reference categories for categorical predictors were female sex assigned at birth, secondary education, BMI < 25.0 kg/m^2^, and daily social media use of ≤1 h/day.

Regression results were reported as unstandardized regression coefficients (*B*) with 95% confidence intervals (CIs) and *p* values. For each multivariable model, the analytical sample size (*n*), coefficient of determination (R^2^), and adjusted R^2^ were reported.

Multicollinearity among predictors in the multivariable models was assessed using the variance inflation factor (VIF), with values <5 considered acceptable. Residual normality was evaluated using the Shapiro–Wilk test, and homoscedasticity was assessed using the Breusch–Pagan test.

Missing data were not imputed. Each regression model was fitted using complete cases for all variables included in that model. Statistical significance was set at *p* < 0.05. All analyses were performed using R statistical software, version 4.6.1 (R Foundation for Statistical Computing, Vienna, Austria) [16].

Given the cross-sectional design, regression results were interpreted as measures of association rather than causal effects.

### 2.6. Data Quality Control

Before the analyses were conducted, a data quality control procedure was implemented. Responses that did not meet the eligibility criteria specified in the screening questions or the predefined data quality criteria, including criteria related to the consistency and plausibility of the responses, were excluded from the analyses.

The data quality control procedure included an attention-check question placed in the introductory section of the questionnaire to assess respondent attentiveness.

### 2.7. Ethical Considerations

The study was conducted in accordance with the principles of the Declaration of Helsinki. The study protocol was approved by the Bioethics Committee of the University of Rzeszow, Collegium Medicum, University of Rzeszow, Rzeszow, Poland, on 29 May 2026 (decision no. 46/05/2026).

Before participation, individuals received information about the purpose and procedures of the study and the principles governing data processing. Access to the questionnaire was granted only after participants had provided electronic informed consent by selecting the appropriate checkbox in the form. The consent page stated that participation was voluntary and anonymous and that participants could withdraw at any stage before submitting the completed questionnaire.

## 3. Results

Of the 317 responses received, 279 questionnaires were included in the final analysis, and 38 were excluded. The participant selection process and reasons for exclusion are summarized in Figure 1. The following sections present the characteristics of the study sample, patterns of exposure to GLP-1-related social media content, exposure to selected narratives and their perceived credibility, perceptions of communication about obesity treatment, attitudes toward obesity pharmacotherapy, evaluations of treatment with GLP-1 receptor agonists, and the results of the regression analyses.

### 3.1. Characteristics of the Study Sample

The analysis included 279 participants. The mean age was 38.14 ± 8.58 years, with a range of 18–60 years. Most participants were female (84.59%). Data required to calculate BMI were available for 268 participants (96.06%). In the total sample, 46.95% of participants had a BMI < 25.0 kg/m^2^, 30.47% had a BMI of 25.0 to <30.0 kg/m^2^, and 18.64% had a BMI ≥ 30.0 kg/m^2^. Ever use of GLP-1 receptor agonists was reported by 21.86% of participants, and 39.43% reported a previous diagnosis of obesity. Most participants (72.04%) reported using social media for at least 1 h/day. Detailed sample characteristics are presented in Table 1.

### 3.2. Exposure to Social Media Content Related to GLP-1 Receptor Agonists

Among participants who met the eligibility criteria, 49.82% reported frequent or very frequent exposure to social media content related to GLP-1 receptor agonists. Occasional exposure was reported by 39.78% of participants, whereas 10.39% reported rare exposure. Because of the eligibility criteria applied, these findings refer exclusively to individuals who reported prior exposure to this type of content and do not reflect the prevalence of exposure among the general population of social media users.

Instagram was the platform most frequently identified as a source of GLP-1-related content (91.04%). Facebook (41.58%), YouTube (34.05%), and TikTok (14.70%) were reported less frequently. Each of the remaining platforms was selected by fewer than 10% of participants.

Short-form video content, such as reels or videos posted on social media, was the predominant format through which participants encountered GLP-1-related content (81.36%). Text-based or graphic posts (58.78%), stories (46.95%), comments under posts or video content (37.99%), and longer-form videos (32.62%) were also frequently reported. Articles or links to external sources were reported by 17.92% of participants, whereas podcasts were reported by 1.43%.

In addition to exposure itself, participants reported various forms of engagement with this type of content. The most frequently reported activities were encountering content in their social media feeds (89.25%) and reading comments posted beneath it (53.41%). Smaller proportions of respondents reported actively searching for information about GLP-1 receptor agonists (22.22%), sharing, saving, or forwarding such content (7.17%), or posting comments (4.30%). Detailed data are presented in Table 2.

### 3.3. Exposure to and Perceived Credibility of GLP-1-Related Narratives

Respondents reported exposure to a range of narratives concerning GLP-1 receptor agonists on social media. The highest frequencies of exposure were observed for statements suggesting that the use of GLP-1 receptor agonists represents a shortcut and a way of avoiding personal effort (46.95% of responses were “often” or “very often”), that individuals using such treatment do so because they lack willpower or discipline (41.94%), and that obesity pharmacotherapy does not constitute genuine treatment of the disease but rather a convenient solution (39.07%).

Participants less frequently reported exposure to narratives suggesting that GLP-1 receptor agonists may lead to cancer development (10.39% reporting exposure “often” or “very often”) or that physicians and pharmaceutical companies promote these medications mainly for financial reasons (24.01%).

For all nine narratives, responses of “completely not credible” and “rather not credible” predominated when combined. The highest combined proportion of “rather credible” and “completely credible” responses was observed for the narrative that GLP-1 receptor agonists often lead to serious health complications (14.70%), followed by the claims that these medications are intended exclusively for people with diabetes and that their use in obesity treatment constitutes misuse (13.98%) and that their use represents a shortcut and avoidance of personal effort (12.19%).

Across the nine credibility items, “difficult to assess” accounted for 518 of the 2511 item-level responses (20.63%). The proportion of these responses across individual items ranged from 10.75% to 30.11% and was highest for the narrative concerning cancer development. The detailed response distributions are presented in Table 3.

### 3.4. Perceptions of Social Media Communication About Obesity Treatment

Most participants agreed with statements indicating that social media communication about obesity treatment was judgmental, stigmatizing and made calm and factual discussion more difficult.

Overall, 69.89% of respondents agreed that the content about GLP-1 receptor agonists published on social media was judgmental toward people living with obesity. A similar proportion of participants (70.97%) agreed that obesity treatment is often presented in a stigmatizing manner. The same proportion agreed that social media discussions about obesity pharmacotherapy make calm and factual discussion of the topic more difficult.

In addition, 63.80% of participants agreed that this type of communication may intensify feelings of shame related to body weight. At the same time, 50.90% agreed that they rarely encountered messages supporting people receiving treatment for obesity. Detailed data are presented in Table 4.

### 3.5. Attitudes Toward Obesity Pharmacotherapy and GLP-1 Treatment Evaluations

Regarding attitudes toward obesity pharmacotherapy, most respondents disagreed with statements that the use of GLP-1 receptor agonists represents “taking a shortcut” (75.99%) or is primarily attributable to a lack of willpower (76.70%). In addition, 65.23% of participants disagreed that treating obesity with medication is less valuable than lifestyle modification without pharmacotherapy.

At the same time, 67.03% of participants agreed that pharmacological treatment of obesity is less socially accepted than obesity treatment without pharmacotherapy. Most respondents disagreed that discussing obesity pharmacotherapy made them feel uncomfortable (80.29%). If medically indicated, 72.40% of respondents agreed that they would consider obesity pharmacotherapy.

Respondents generally evaluated obesity treatment with GLP-1 receptor agonists favorably. Overall, 76.70% of participants agreed that GLP-1 receptor agonists are an effective treatment for obesity, 54.84% agreed that they are safe, and 73.84% considered them a legitimate treatment for the disease. In addition, 70.97% of respondents agreed that they would trust a physician who recommended such treatment. Detailed data are presented in Table 5.

### 3.6. Internal Consistency and Regression Analyses

Internal consistency was assessed for all five composite scores. Cronbach’s α and McDonald’s omega total were 0.884 and 0.922 for narrative exposure, 0.893 and 0.929 for perceived narrative credibility, 0.877 and 0.905 for perceptions of social media communication, 0.592 and 0.800 for attitudes toward obesity pharmacotherapy, and 0.905 and 0.929 for the evaluation of GLP-1 receptor agonists as an obesity treatment, respectively. All estimates were based on data from 279 participants and are summarized in Supplementary Table S1.

In the univariable models, higher narrative exposure was associated with more favorable views of GLP-1 receptor agonists as an obesity treatment (*B* = 0.110; 95% CI: 0.054 to 0.167; *p* < 0.001) and stronger agreement with negative appraisals of social media communication (*B* = 0.236; 95% CI: 0.163 to 0.308; *p* < 0.001). Higher perceived narrative credibility was associated with less favorable views of GLP-1 receptor agonists as an obesity treatment (*B* = −0.319; 95% CI: −0.368 to −0.271; *p* < 0.001) and greater endorsement of negative statements about obesity pharmacotherapy (*B* = 0.401; 95% CI: 0.350 to 0.452; *p* < 0.001).

After adjustment for the other variables included in each model, higher perceived narrative credibility remained associated with less favorable views of GLP-1 receptor agonists as an obesity treatment (*B* = −0.311; 95% CI: −0.359 to −0.263; *p* < 0.001) and greater endorsement of negative statements about obesity pharmacotherapy (*B* = 0.401; 95% CI: 0.350 to 0.453; *p* < 0.001). Higher narrative exposure remained associated with more favorable views of GLP-1 receptor agonists as an obesity treatment (*B* = 0.072; 95% CI: 0.026 to 0.118; *p* = 0.002) and stronger agreement with negative appraisals of social media communication (*B* = 0.235; 95% CI: 0.161 to 0.309; *p* < 0.001), but was not associated with the attitudes toward obesity pharmacotherapy score (*B* = 0.004; 95% CI: −0.046 to 0.054; *p* = 0.869).

Compared with participants with a BMI < 25.0 kg/m^2^, those with a BMI of 30.0 to <35.0 kg/m^2^ held more favorable views of GLP-1 receptor agonists as an obesity treatment (*B* = 1.260; 95% CI: 0.174 to 2.345; *p* = 0.023) and had lower attitudes toward obesity pharmacotherapy scores, indicating weaker endorsement of negative statements about such treatment (*B* = −1.550; 95% CI: −2.741 to −0.359; *p* = 0.011). Each additional year of age was associated with a lower score for perceptions of social media communication, indicating weaker agreement with negative appraisals of such communication (*B* = −0.072; 95% CI: −0.139 to −0.006; *p* = 0.033).

In the univariable model, participants reporting 1–2 h/day of social media use had higher scores on the scale evaluating GLP-1 receptor agonists as an obesity treatment than those reporting ≤1 h/day (*B* = 1.083; 95% CI: 0.027 to 2.139; *p* = 0.044). However, this association was attenuated after adjustment for the other variables included in the model and was no longer statistically significant (*B* = 0.729; 95% CI: −0.101 to 1.560; *p* = 0.085). No adjusted difference was observed between participants reporting >2 h/day and those reporting ≤1 h/day (*B* = 0.438; 95% CI: −0.450 to 1.326; *p* = 0.332).

Model diagnostics provided no evidence of multicollinearity (all variance inflation factors <5), departure from normality of the residuals (all Shapiro–Wilk test *p*-values > 0.05), or heteroscedasticity (all Breusch–Pagan test *p*-values > 0.05). Model sample sizes, model fit statistics (R^2^ and adjusted R^2^), and selected multivariable coefficients are presented in Table 6. Complete univariable and multivariable results are provided in Supplementary Tables S2 and S3, respectively.

## 4. Discussion

This exploratory cross-sectional study examined whether exposure to social media narratives concerning GLP-1 receptor agonists and the credibility attributed to these narratives were associated with perceptions of obesity pharmacotherapy. In the multivariable models, greater perceived credibility of the analyzed narratives was associated with both less favorable evaluations of GLP-1 receptor agonists as an obesity treatment and greater endorsement of negative statements about obesity pharmacotherapy. Greater narrative exposure, in contrast, was associated with more favorable evaluations of GLP-1 receptor agonist treatment and stronger agreement with negative appraisals of social media communication, but it was not associated with attitudes toward obesity pharmacotherapy after adjustment for the other variables. This outcome-specific pattern highlights the need to distinguish between the frequency with which narratives are encountered and the credibility attributed to them. The positive association between narrative exposure and GLP-1 treatment evaluation should not be interpreted as evidence that exposure improved treatment perceptions. It may instead reflect prior interest in the treatment, information-seeking behavior, or selective attention, although these mechanisms were not assessed in the present study. Daily social media use was associated with GLP-1 treatment evaluation only in the univariable analysis: the difference between participants reporting 1–2 h/day and those reporting ≤1 h/day was attenuated after adjustment, and no adjusted difference was observed for >2 h/day. The findings therefore do not indicate an independent or graded association between daily time spent using social media and treatment evaluation. Because exposure, perceived credibility, and treatment-related attitudes were assessed concurrently, the direction of the observed associations cannot be determined. Experimental research conducted in other social media contexts indicates that source credibility can modify how misinformation and corrective messages are evaluated [17]. This evidence supports distinguishing perceived credibility from exposure but does not establish a causal relationship in the present study.

These findings may be relevant to contemporary obesity management because GLP-1 receptor agonists are increasingly discussed not only as pharmacological agents, but also as socially contested treatments [9]. In clinical practice, treatment decisions are made in an information environment in which patients may encounter messages that question the legitimacy, safety, or social acceptability of pharmacotherapy. The present findings are consistent with experimental evidence showing that people described as losing weight with a GLP-1 receptor agonist were evaluated more negatively than those described as losing weight through diet and exercise, partly because pharmacotherapy was perceived as a “shortcut” [18,19]. In another experiment, exposure to GLP-1-assisted weight loss increased overall support for policies expanding access to these medications, but shortcut beliefs constituted an opposing indirect pathway associated with lower policy support [20]. Although the present study did not assess treatment initiation, adherence, or clinical outcomes, the observed associations suggest that perceived narrative credibility warrants prospective investigation as a factor potentially relevant to treatment appraisal.

The findings should be considered in the context of broader concerns regarding the quality of health information disseminated in digital environments. Health and nutrition misinformation has been recognized as an important public health issue, and social media has become one of the main channels through which both reliable information and incomplete, oversimplified, or misleading content are disseminated [14]. The quality of nutrition information published online remains highly variable, and a substantial proportion of available materials does not meet basic standards of quality or factual accuracy [15]. An additional challenge is that many users experience difficulty in assessing the credibility of health information and distinguishing content consistent with current scientific evidence from false or misleading information [21]. A related pattern of uncertainty was observed in the present study: “difficult to assess” accounted for 20.63% of responses across the credibility items and ranged from 10.75% to 30.11% across individual narratives, particularly those concerning cancer risk, serious complications, and lifelong treatment. Such responses may reflect uncertainty or difficulty evaluating conflicting information rather than endorsement of these narratives; however, the reasons underlying them were not assessed. Because this response represented the midpoint of the scale, the composite credibility score may partly capture uncertainty and should be interpreted accordingly. Recent content analyses provide complementary evidence concerning the quality and completeness of GLP-1-related information on social media. In an analysis of Spanish-speaking TikTok videos, the mean DISCERN score was only 29.8 out of 75, 71% of videos did not mention any treatment risk, and 85% framed obesity as an individual problem [22]. A separate analysis of 250 posts across five social media platforms identified incorrect pharmacological statements in 10% of posts and substantial omissions concerning approved indications, adverse effects, and mechanisms of action [23]. These studies evaluated selected samples of content from specific platforms rather than users’ perceptions and therefore cannot establish what individual users encountered. Nevertheless, they document the presence of incomplete and inaccurate GLP-1-related information in the sampled content.

The relevance of these observations may be reinforced by the way social media platforms operate. Evidence from contexts outside health communication indicates that false news can spread farther and faster than truthful news, be perceived as more novel, and elicit different emotional responses [24]. At the same time, health and nutrition information is frequently published without reference to scientific sources or presented in ways that make independent assessment of its reliability difficult [25]. As a result, users navigate an information environment in which judgments of credibility may depend not only on the quality of the evidence presented, but also on the appeal of the narrative, the level of engagement generated by the content, and the perceived credibility of the source [17,24,25]. These mechanisms represent plausible interpretations of the observed pattern, but they were not measured directly. In particular, the study did not assess platform algorithms, emotional engagement, source characteristics, message repetition, or participants’ prior agreement with the narratives. The present results therefore do not establish which mechanisms account for the different associations observed for narrative exposure and perceived credibility.

The nature of the narratives most frequently encountered by participants in our study also warrants attention. The most frequently reported messages portrayed GLP-1 receptor agonist use as a “shortcut,” attributed its use to a lack of willpower, or questioned its legitimacy as a genuine treatment for obesity. By contrast, narratives concerning a potential risk of cancer were among the least frequently reported. Taken together, this pattern suggests that moralized narratives concerning effort, willpower, and treatment legitimacy were prominent in participants’ recalled social media exposure, although clinically framed messages concerning treatment risks were also reported.

This pattern is consistent with the broader weight-stigma literature, in which beliefs about the controllability of body weight and personal responsibility contribute to judgments about effort, character, and deservingness [26,27]. Together with GLP-1-specific experimental findings [18,19], these observations suggest that stigmatizing judgments may extend beyond body size to the perceived legitimacy of the treatment method used. Despite a growing body of evidence indicating that body weight regulation results from complex interactions among biological, environmental, psychological, and social factors [28,29], people with obesity continue to encounter the belief that the disease is largely a consequence of insufficient self-control, lack of motivation, or inappropriate lifestyle choices [26,27]. Overall, 69.89% of participants perceived social media content concerning obesity treatment as judgmental toward people with obesity, 70.97% considered such content stigmatizing, and 63.80% indicated that this type of communication may intensify feelings of shame related to body weight. These percentages describe participants’ perceptions within the selected study sample and should not be interpreted as estimates of the actual prevalence of stigmatizing GLP-1-related content on social media. Weight stigma may be associated with greater psychological distress, avoidance of healthcare, poorer experiences within healthcare systems, and a reduced likelihood of seeking professional treatment [26,27].

These observations may be particularly relevant in the context of contemporary obesity care, in which pharmacotherapy is an established component of comprehensive treatment [4]. GLP-1 receptor agonists have been shown to produce clinically meaningful weight loss, together with reductions in BMI and waist circumference [6,7]. Current recommendations also emphasize that GLP-1-based treatment should be accompanied by appropriate nutritional support, physical activity, and behavioral interventions [12,13]. Framing pharmacotherapy as an alternative to personal effort may instead position medication and dietary or lifestyle change as competing approaches, potentially obscuring their complementary roles in comprehensive obesity care. Whether this type of framing is associated with treatment decisions, adherence, or interactions with healthcare professionals was not assessed in the present study.

The findings suggest that communication strategies addressing misleading or oversimplified narratives about obesity treatment may need to consider both the availability of reliable information and how users assess the credibility of sources and claims [17]. This may be particularly important in social media environments, which are widely used for health information seeking [10,11]. Healthcare professionals, scientific organizations, and public health institutions may contribute by providing accessible evidence-based information and supporting critical evaluation of the evidence, expertise, and potential conflicts of interest underlying health claims [17,21]. However, the effectiveness of such approaches cannot be inferred from the present cross-sectional study. Future longitudinal and experimental studies should test whether interventions targeting source evaluation and critical appraisal improve understanding of obesity pharmacotherapy and affect subsequent treatment-related attitudes or decisions.

## 5. Strengths and Limitations

To the best of our knowledge, this is one of the first studies to examine the associations between exposure to social media content concerning GLP-1 receptor agonists, the credibility attributed to such content, and perceptions of obesity pharmacotherapy. An additional strength of the study was the simultaneous assessment of both exposure to the narratives under investigation and their perceived credibility, which enabled a more comprehensive evaluation of the potential relationships among information exposure, perceived credibility, and attitudes toward obesity pharmacotherapy. The study also covered a broad range of domains, including perceptions of communication about obesity treatment, attitudes toward pharmacotherapy, and evaluations of treatment with GLP-1 receptor agonists.

The findings should, however, be interpreted in light of several limitations. Because of the cross-sectional design, the direction of the observed associations cannot be determined, and causal inferences cannot be made. The study used a convenience sample recruited through public social media posts, including posts published on accounts managed by members of the research team and in relevant thematic groups, with further dissemination through sharing. This recruitment strategy may have preferentially reached individuals with a greater interest in health, nutrition, obesity, or pharmacotherapy. It may also limit the generalizability of the findings beyond users reached through these channels. In addition, women constituted 84.6% of the sample; therefore, the results primarily reflect perceptions within a predominantly female group and should be extrapolated to men with caution.

All data were self-reported and may therefore be affected by recall and reporting biases. The questionnaire was developed specifically for this exploratory study and had not undergone prior formal validation. Although four composite scores demonstrated high internal consistency, the attitudes toward obesity pharmacotherapy score yielded a Cronbach’s alpha of 0.592 and a McDonald’s omega total of 0.800 (Supplementary Table S1). Given the discrepancy between these estimates and the absence of prior validation, findings involving this composite score should be interpreted cautiously. All composite scores should be regarded as exploratory and require further psychometric evaluation in independent samples.

Prior exposure to social media content concerning GLP-1 receptor agonists was a mandatory eligibility criterion, consistent with the study objective of examining perceptions among individuals who had encountered such content. Consequently, the study did not include an unexposed comparison group, and the findings cannot be used to estimate the prevalence of exposure to GLP-1-related content in the general population. Moreover, the study assessed participants’ recalled exposure to predefined narratives and the credibility attributed to them. It did not directly analyze the content that participants had encountered or independently verify its sources, context, or factual accuracy. The findings should therefore not be interpreted as estimates of the prevalence or effects of objectively verified misinformation.

Future studies should include more diverse and representative samples with a more balanced representation of women and men, use longitudinal or experimental designs, employ validated measurement instruments, and, where feasible, combine self-reported exposure with direct assessment of social media content.

## 6. Conclusions

The findings indicate that the credibility attributed to social media narratives may be relevant to participants’ perceptions of obesity pharmacotherapy, including their evaluations of treatment with GLP-1 receptor agonists. However, because the study was cross-sectional, the direction of the observed associations cannot be determined, and causal relationships cannot be established. The findings should also be interpreted in relation to the self-reported nature of the data and the predominantly female convenience sample recruited through social media.

Despite these limitations, the findings highlight the importance of addressing not only the availability of reliable information, but also how users evaluate the credibility of health content in digital environments. Communication concerning obesity care should present pharmacotherapy, nutritional support, physical activity, and behavioral interventions as complementary components of comprehensive treatment rather than as competing alternatives. Longitudinal and experimental studies are needed to identify the factors shaping perceived credibility and to determine whether changes in credibility are associated with subsequent changes in treatment-related attitudes and decisions.

## Figures and Tables

**Table 1:** Characteristics of the study sample (*n* = 279).

Characteristic	Value
Age, years	38.14 ± 8.58
Age range, years	18–60
**Sex assigned at birth**	
Female	236 (84.59)
Male	43 (15.41)
**BMI category, kg/m^2^**	
<25.0	131 (46.95)
25.0 to <30.0	85 (30.47)
30.0 to <35.0	32 (11.47)
35.0 to <40.0	11 (3.94)
≥40.0	9 (3.23)
Missing BMI data	11 (3.94)
**Educational attainment**	
Secondary	45 (16.13)
Bachelor’s or engineering degree	59 (21.15)
Master’s degree	166 (59.50)
Doctoral degree	6 (2.15)
Prefer not to answer	3 (1.08)
**Daily time spent using social media**	
<30 min	4 (1.43)
30–60 min	74 (26.52)
1–2 h	113 (40.50)
2–4 h	72 (25.81)
>4 h	16 (5.73)
Ever use of GLP-1 receptor agonists for obesity treatment	
Yes	61 (21.86)
No	215 (77.06)
Prefer not to answer	3 (1.08)
**Self-reported previous diagnosis of obesity**	
No	151 (54.12)
Yes	110 (39.43)
Do not know	15 (5.38)
Prefer not to answer	3 (1.08)

Data are presented as mean ± SD, range, or *n* (%), as appropriate. Percentages were calculated using the total sample (*n* = 279) as the denominator. BMI was calculated from self-reported height and body weight. Abbreviations: BMI, body mass index; SD, standard deviation.

**Table 2:** Exposure to GLP-1-Related Content on Social Media.

Variable	*n* (%)
**Frequency of exposure to GLP-1-related content**	
Very often	31 (11.11)
Often	108 (38.71)
Sometimes	111 (39.78)
Rarely	29 (10.39)
**Platforms where participants encountered GLP-1-related content ^1^**	
Instagram	254 (91.04)
Facebook	116 (41.58)
YouTube	95 (34.05)
TikTok	41 (14.70)
Internet forums	23 (8.24)
X/Twitter	12 (4.30)
Spotify	3 (1.08)
Tumblr	2 (0.72)
Reddit	1 (0.36)
**Formats of encountered content ^1^**	
Short-form video (e.g., reels, TikTok)	227 (81.36)
Text or image posts	164 (58.78)
Stories	131 (46.95)
Comments under posts or videos	106 (37.99)
Longer video materials	91 (32.62)
Articles or external links	50 (17.92)
Podcasts	4 (1.43)
**User engagement related to GLP-1-related content ^1^**	
Saw content in personal feed	249 (89.25)
Read comments under such content	149 (53.41)
Searched for information independently	62 (22.22)
Shared, saved or forwarded such content	20 (7.17)
Commented on such content	12 (4.30)

^1^ Multiple-response item; percentages do not sum to 100%.

**Table 3:** Exposure to Specific Narratives Concerning GLP-1 Receptor Agonists and Their Perceived Credibility.

Panel A. Exposure to Specific Narratives Concerning GLP-1 Receptor Agonists					
Statement	Never	Rarely	Sometimes	Often	Very Often
	*n* (%)	*n* (%)	*n* (%)	*n* (%)	*n* (%)
Using GLP-1 analogues is a shortcut and avoidance of personal effort	11 (3.94)	36 (12.90)	101 (36.20)	99 (35.48)	32 (11.47)
GLP-1 treatment is not real treatment of disease, only a convenient solution	21 (7.53)	59 (21.15)	90 (32.26)	83 (29.75)	26 (9.32)
People use GLP-1 because they lack willpower or discipline	21 (7.53)	51 (18.28)	90 (32.26)	89 (31.90)	28 (10.04)
Weight always returns after discontinuation regardless of other actions	43 (15.41)	58 (20.79)	72 (25.81)	73 (26.16)	33 (11.83)
GLP-1 should be used lifelong, and effects cannot be maintained without it	79 (28.32)	68 (24.37)	57 (20.43)	55 (19.71)	20 (7.17)
GLP-1 often leads to serious health complications	31 (11.11)	69 (24.73)	75 (26.88)	77 (27.60)	27 (9.68)
GLP-1 may lead to cancer development	146 (52.33)	71 (25.45)	33 (11.83)	22 (7.89)	7 (2.51)
Physicians and pharmaceutical companies promote GLP-1 receptor agonists mainly for financial reasons	81 (29.03)	73 (26.16)	58 (20.79)	46 (16.49)	21 (7.53)
GLP-1 is intended only for diabetes, and obesity treatment is misuse	36 (12.90)	67 (24.01)	95 (34.05)	59 (21.15)	22 (7.89)
**Panel B. Perceived credibility of specific narratives concerning GLP-1 receptor agonists**					
**Statement**	**Completely not credible**	**Rather not credible**	**Difficult to assess**	**Rather credible**	**Completely credible**
	***n* (%)**	***n* (%)**	***n* (%)**	***n* (%)**	***n* (%)**
Using GLP-1 analogues is a shortcut and avoidance of personal effort	124 (44.44)	75 (26.88)	46 (16.49)	26 (9.32)	8 (2.87)
GLP-1 treatment is not real treatment of disease, only a convenient solution	151 (54.12)	65 (23.30)	30 (10.75)	26 (9.32)	7 (2.51)
People use GLP-1 because they lack willpower or discipline	129 (46.24)	71 (25.45)	48 (17.20)	27 (9.68)	4 (1.43)
Weight always returns after discontinuation regardless of other actions	91 (32.62)	100 (35.84)	61 (21.86)	23 (8.24)	4 (1.43)
GLP-1 should be used lifelong, and effects cannot be maintained without it	112 (40.14)	81 (29.03)	69 (24.73)	14 (5.02)	3 (1.08)
GLP-1 often leads to serious health complications	71 (25.45)	87 (31.18)	80 (28.67)	33 (11.83)	8 (2.87)
GLP-1 may lead to cancer development	101 (36.20)	81 (29.03)	84 (30.11)	11 (3.94)	2 (0.72)
Physicians and pharmaceutical companies promote GLP-1 receptor agonists mainly for financial reasons	111 (39.78)	79 (28.32)	59 (21.15)	22 (7.89)	8 (2.87)
GLP-1 is intended only for diabetes, and obesity treatment is misuse	131 (46.95)	68 (24.37)	41 (14.70)	24 (8.60)	15 (5.38)

Data are presented as *n* (%). Exposure and credibility were assessed using separate five-point Likert scales.

**Table 4:** Perceptions of Social Media Communication Regarding Obesity Treatment.

Statement	Strongly Disagree	Rather Disagree	Neither Agree nor Disagree	Rather Agree	Strongly Agree
	*n* (%)	*n* (%)	*n* (%)	*n* (%)	*n* (%)
Content about GLP-1 receptor agonists on social media is often judgmental toward people living with obesity	11 (3.94)	39 (13.98)	34 (12.19)	105 (37.63)	90 (32.26)
On social media, obesity treatment is often presented in a stigmatizing way	11 (3.94)	35 (12.54)	35 (12.54)	117 (41.94)	81 (29.03)
Discussions about obesity pharmacotherapy on social media make calm and factual discussion of the topic more difficult	15 (5.38)	30 (10.75)	36 (12.90)	103 (36.92)	95 (34.05)
On social media, I rarely encounter messages supporting people receiving treatment for obesity	27 (9.68)	73 (26.16)	37 (13.26)	88 (31.54)	54 (19.35)
Communication about GLP-1 receptor agonists on social media may increase shame related to body weight	24 (8.60)	31 (11.11)	46 (16.49)	93 (33.33)	85 (30.47)

Data are presented as *n* (%). Responses were assessed using a five-point Likert scale.

**Table 5:** Attitudes Toward Obesity Pharmacotherapy and Evaluation of Treatment With GLP-1 Receptor Agonists.

Statement	Strongly Disagree	Rather Disagree	Neither Agree nor Disagree	Rather Agree	Strongly Agree
	*n* (%)	*n* (%)	*n* (%)	*n* (%)	*n* (%)
**Attitudes toward obesity pharmacotherapy**					
Using GLP-1 receptor agonists in obesity treatment is “taking a shortcut”	145 (51.97)	67 (24.01)	33 (11.83)	28 (10.04)	6 (2.15)
People with obesity who use GLP-1 receptor agonists do so mainly because they lack willpower	140 (50.18)	74 (26.52)	32 (11.47)	29 (10.39)	4 (1.43)
Obesity treatment with medication is less valuable than lifestyle modification without pharmacotherapy	123 (44.09)	59 (21.15)	42 (15.05)	34 (12.19)	21 (7.53)
Talking about obesity pharmacotherapy causes me discomfort	181 (64.87)	43 (15.41)	29 (10.39)	20 (7.17)	6 (2.15)
Obesity treatment with medication is less socially accepted than obesity treatment without pharmacotherapy	29 (10.39)	25 (8.96)	38 (13.62)	95 (34.05)	92 (32.97)
If medically indicated, I would be willing to consider obesity pharmacotherapy	30 (10.75)	19 (6.81)	28 (10.04)	62 (22.22)	140 (50.18)
**Evaluation of Treatment With GLP-1 Receptor Agonists**					
Using GLP-1 receptor agonists is an effective method of obesity treatment	6 (2.15)	8 (2.87)	51 (18.28)	103 (36.92)	111 (39.78)
Using GLP-1 receptor agonists is a safe method of obesity treatment	6 (2.15)	28 (10.04)	92 (32.97)	89 (31.90)	64 (22.94)
Obesity treatment with GLP-1 receptor agonists should be considered a legitimate treatment for the disease	12 (4.30)	17 (6.09)	44 (15.77)	89 (31.90)	117 (41.94)
I trust a physician who recommends obesity treatment with GLP-1 receptor agonists	9 (3.23)	21 (7.53)	51 (18.28)	85 (30.47)	113 (40.50)

Data are presented as *n* (%). Responses were assessed using a five-point Likert scale.

**Table 6:** Model Fit and Selected Coefficients From the Multivariable Linear Regression Models.

Outcome	Model Sample Size (*n*)	R^2^	Adjusted R^2^	Predictor	*B*	95% CI	*p*-Value
Evaluation of GLP-1 receptor agonists as an obesity treatment	268	0.465	0.438	Narrative exposure score	0.072	0.026 to 0.118	0.002
				Perceived narrative credibility score	−0.311	−0.359 to −0.263	<0.001
				BMI: 30.0 to <35.0 vs. <25.0 kg/m^2^	1.260	0.174 to 2.345	0.023
				Social media use: 1–2 vs. ≤1 h/day	0.729	−0.101 to 1.560	0.085
				Social media use: >2 vs. ≤1 h/day	0.438	−0.450 to 1.326	0.332
Attitudes toward obesity pharmacotherapy	268	0.515	0.492	Narrative exposure score	0.004	−0.046 to 0.054	0.869
				Perceived narrative credibility score	0.401	0.350 to 0.453	<0.001
				BMI: 30.0 to <35.0 vs. <25.0 kg/m^2^	−1.550	−2.741 to −0.359	0.011
Perceptions of social media communication	279	0.163	0.135	Narrative exposure score	0.235	0.161 to 0.309	<0.001
				Age, years	−0.072	−0.139 to −0.006	0.033

Abbreviations: *B*, unstandardized regression coefficient; BMI, body mass index; CI, confidence interval. Model sample size (*n*) denotes the number of participants included in the complete multivariable model. Higher scores for the three outcomes indicate, respectively, more favorable views of GLP-1 receptor agonists as an obesity treatment, greater endorsement of negative statements about obesity pharmacotherapy, and stronger agreement with negative appraisals of social media communication. The table presents the principal explanatory variables, daily social media use comparisons for the model evaluating GLP-1 receptor agonists as an obesity treatment, and other covariates with *p* < 0.05 in the adjusted models. Complete univariable and multivariable results are provided in Supplementary Tables S2 and S3.

## Data Availability

The datasets generated and analyzed during the current study are available from the corresponding author upon reasonable request. The datasets are not publicly available because participants did not provide consent for public data sharing.

## Supplementary Materials

The following supporting information can be downloaded at: https://www.mdpi.com/article/10.3390/nu18162718/s1, Supplementary Questionnaire S1: English Translation of the Original Polish Questionnaire; Supplementary Table S1: Internal Consistency of the Composite Scores Used in the Regression Analyses; Supplementary Table S2: Univariable Linear Regression Analyses of Factors Associated with Evaluation of GLP-1 Receptor Agonists as an Obesity Treatment, Attitudes Toward Obesity Pharmacotherapy, and Perceptions of Social Media Communication About Obesity Treatment; Supplementary Table S3: Multivariable Linear Regression Analyses of Factors Associated with Evaluation of GLP-1 Receptor Agonists as an Obesity Treatment, Attitudes Toward Obesity Pharmacotherapy, and Perceptions of Social Media Communication About Obesity Treatment.

## Abbreviations

The following abbreviations are used in this manuscript:

BMI	Body mass index
CI	Confidence interval
GLP-1	Glucagon-like peptide-1
WHO	World Health Organization
