# Supplementary material for: Perceived Credibility of Social Media Narratives About GLP-1 Receptor Agonists and Acceptance of Integrated Obesity Care: Implications for Nutrition and Lifestyle Support
Source: Nutrients. 2026 Aug 20;18(16):2718. doi: 10.3390/nu18162718 (PMC13516904; doi:10.3390/nu18162718)
Supplement: Supplementary file 1 [file nutrients-18-02718-s001.zip › nutrients-4465761-supplementary.pdf]

Supplementary Materials

Article title: Perceived Credibility of Social Media Narratives About GLP-1 Receptor Agonists and Acceptance of Integrated Obesity Care: Implications for Nutrition and Lifestyle Support

Translation and reporting note

The original Polish questionnaire was developed specifically for this exploratory study and had not undergone prior formal validation. Internal consistency of the composite scores in the present sample is reported in Supplementary Table S1. The English translation presented below was prepared for transparency and reproducibility, was not used during participant recruitment or data collection, and has not been independently validated.

For consistency with the manuscript, the Polish term corresponding to "GLP-1 analogues" is rendered here as "GLP-1 receptor agonists". Examples used in the original questionnaire included Ozempic and Wegovy. Exposure-related items referred to content encountered on social media during the preceding 3 months.

Supplementary Questionnaire S1. English Translation of the Original Polish Questionnaire

Survey title

The Role of Social Media Communication in Shaping Attitudes Toward Pharmacotherapy for Obesity with GLP-1 Receptor Agonists

Study information

This survey concerns content related to GLP-1 receptor agonists (e.g., Ozempic, Wegovy) encountered on social media during the preceding 3 months and its possible influence on attitudes toward obesity treatment.

This is an anonymous scientific study. Completion takes approximately 5-7 minutes.

Section 1. Preliminary Information, Consent, and Eligibility Screening

1. Do you provide informed consent to participate in this anonymous scientific study?

- ☐ Yes
- ☐ No

2. Please read this question carefully and select "Rather disagree".

- ☐ Strongly disagree
- ☐ Rather disagree
- ☐ Neither agree nor disagree
- ☐ Rather agree
- ☐ Strongly agree

3. Please provide your age (in years):

4. Do you use social media at least once per week?

- ☐ Yes
- ☐ No

This study concerns content encountered on social media. If "No" is selected, the form is ended.

5. During the preceding 3 months, have you encountered content on social media regarding the use of GLP-1 receptor agonists (e.g., Ozempic, Wegovy) in the context of weight loss or obesity treatment?

- ☐ Yes

☐ No

☐ I do not know

Only individuals who declared exposure to such content on social media during the preceding 3 months qualified for the further part of the survey.

Section 2. Exposure to Content About GLP-1 Receptor Agonists on Social Media

6. During the preceding 3 months, how often have you encountered content regarding GLP-1 receptor agonists in the context of weight loss or obesity treatment on social media?

☐ Never

☐ Rarely

☐ Sometimes

☐ Often

☐ Very often

7. During the preceding 3 months, on which social media platforms have you encountered content regarding GLP-1 receptor agonists?

☐ Facebook

☐ Instagram

☐ TikTok

☐ YouTube

☐ X (Twitter)

☐ Internet forums

☐ Other: __________

Select all that apply.

8. During the preceding 3 months, in what form have you most often encountered content regarding GLP-1 receptor agonists on social media?

☐ Short-form videos (e.g., reels, TikTok)

☐ Longer video materials

☐ Text or image posts

☐ Comments under posts or videos

☐ Stories

☐ Articles or external links

☐ Other: __________

Select all that apply.

9. During the preceding 3 months, which forms of activity have you encountered in relation to content about GLP-1 receptor agonists on social media?

☐ I saw such content in my feed

☐ I searched for information on this topic

☐ I read comments under such content

☐ I commented on such content

☐ I shared, saved, or forwarded such content

Select all that apply.

Section 3. Narratives About GLP-1 Receptor Agonists Encountered on Social Media

10. During the preceding 3 months, how often have you encountered content on social media suggesting that:

Select one response in each row. Response scale: Never, Rarely, Sometimes, Often, Very often.

Item	Statement
a	using GLP-1 receptor agonists is a shortcut and avoidance of personal effort
b	obesity treatment with GLP-1 receptor agonists is not a genuine treatment of disease, but only a convenient solution
c	people using GLP-1 receptor agonists do so because they lack willpower or discipline
d	after discontinuation of GLP-1 receptor agonists, body weight always returns, regardless of other actions
e	GLP-1 receptor agonists must be used lifelong, and without them the effects cannot be maintained
f	using GLP-1 receptor agonists often leads to serious health complications
g	using GLP-1 receptor agonists may lead to cancer development
h	physicians and pharmaceutical companies promote GLP-1 receptor agonists mainly for financial reasons
i	GLP-1 receptor agonists are intended only for people with diabetes, and their use in obesity treatment is misuse

Section 4. Assessment of the Credibility of Narratives About GLP-1 Receptor Agonists

11. How credible do the following narratives about GLP-1 receptor agonists encountered on social media during the preceding 3 months seem to you?

Select one response in each row. Response scale: Completely not credible, Rather not credible, Difficult to assess, Rather credible, Completely credible.

Item	Statement
a	using GLP-1 receptor agonists is a shortcut and avoidance of personal effort
b	obesity treatment with GLP-1 receptor agonists is not a genuine treatment of disease, but only a convenient solution
c	people using GLP-1 receptor agonists do so because they lack willpower or discipline
d	after discontinuation of GLP-1 receptor agonists, body weight always returns, regardless of other actions
e	GLP-1 receptor agonists must be used lifelong, and without them the effects cannot be maintained
f	using GLP-1 receptor agonists often leads to serious health complications
g	using GLP-1 receptor agonists may lead to cancer development
h	physicians and pharmaceutical companies promote GLP-1 receptor agonists mainly for financial reasons
i	GLP-1 receptor agonists are intended only for people with diabetes, and their use in obesity treatment is misuse

Section 5. Perceptions of Communication About Obesity Treatment on Social Media

12. To what extent do you agree with the following statements?

Select one response in each row. Response scale: Strongly disagree, Rather disagree, Neither agree nor disagree, Rather agree, Strongly agree.

Item	Statement
a	Content about GLP-1 receptor agonists on social media often has a judgmental tone toward people living with obesity.
b	On social media, obesity treatment is often presented in a stigmatizing way.
c	Discussions about obesity pharmacotherapy on social media make calm and factual conversation on this topic more difficult.
d	On social media, I rarely encounter messages supporting people treated for obesity.

Item	Statement
e	Communication around GLP-1 receptor agonists on social media may increase shame related to body weight.

Section 6. Attitudes Toward Obesity Pharmacotherapy

13. To what extent do you agree with the following statements?

Select one response in each row. Response scale: Strongly disagree, Rather disagree, Neither agree nor disagree, Rather agree, Strongly agree.

Item	Statement
a	Using GLP-1 receptor agonists in obesity treatment is “taking a shortcut”.
b	People living with obesity who use GLP-1 receptor agonists do so mainly because they lack willpower.
c	Obesity treatment with medication is less valuable than lifestyle change without pharmacotherapy.
d	Talking about obesity pharmacotherapy causes me discomfort.
e	Obesity treatment with medication is socially less accepted than obesity treatment without pharmacotherapy.
f	If medically indicated, I would be willing to consider obesity pharmacotherapy.

Section 7. Evaluation of Obesity Treatment with GLP-1 Receptor Agonists

14. To what extent do you agree with the following statements?

Select one response in each row. Response scale: Strongly disagree, Rather disagree, Neither agree nor disagree, Rather agree, Strongly agree.

Item	Statement
a	Using GLP-1 receptor agonists is an effective method of obesity treatment.
b	Using GLP-1 receptor agonists is a safe method of obesity treatment.
c	Obesity treatment with GLP-1 receptor agonists should be considered a legitimate treatment of disease.
d	I trust a physician who recommends obesity treatment with GLP-1 receptor agonists.

Section 8. Demographic and Health Data

15. Sex assigned at birth

☐ Female

☐ Male

16. Height (cm):

17. Body weight (kg):

18. Education

☐ Primary education

☐ Vocational education

☐ Secondary education

☐ Bachelor’s or engineering degree

☐ Master’s degree

☐ Doctoral degree

☐ Prefer not to answer

19. How much time per day do you spend using social media?

☐ Less than 30 minutes

☐ 30-60 minutes

- ☐ 1-2 hours
- ☐ 2-4 hours
- ☐ More than 4 hours

20. Have you ever used pharmacotherapy for obesity treatment, including GLP-1 receptor agonists?

- ☐ Yes, I have used GLP-1 receptor agonists
- ☐ Yes, I have used other obesity pharmacotherapy
- ☐ No, I have never used obesity pharmacotherapy
- ☐ Prefer not to answer

21. Have you ever been diagnosed with obesity?

- ☐ Yes
- ☐ No
- ☐ I do not know
- ☐ Prefer not to answer

Supplementary Statistical Tables

The regression results are reported as unstandardized regression coefficients (*B*), 95% confidence intervals (95% CIs), and *p*-values. Model sample sizes (*n*) are also provided. Statistical significance is indicated with an asterisk for $p < 0.05$.

Supplementary Table S1. Internal consistency of the composite scores used in the regression analyses.

Composite score	Number of items	*n*	Cronbach's α	McDonald's omega total
Narrative exposure	9	279	0.884	0.922
Perceived narrative credibility	9	279	0.893	0.929
Perceptions of social media communication	5	279	0.877	0.905
Attitudes toward obesity pharmacotherapy	6	279	0.592	0.800
Evaluation of GLP-1 receptor agonists as an obesity treatment	4	279	0.905	0.929

Abbreviation: α, Cronbach's alpha. *n* denotes the number of participants included in each internal consistency analysis. All estimates were calculated using complete item data.

Supplementary Table S2. Univariable Linear Regression Analyses of Factors Associated With Evaluation of GLP-1 Receptor Agonists as an Obesity Treatment, Attitudes Toward Obesity Pharmacotherapy, and Perceptions of Social Media Communication About Obesity Treatment.

Panel A. Outcome: Evaluation of GLP-1 receptor agonists as an obesity treatment

Predictor	Model sample size (*n*)	*B*	95% CI	*p*-value
Narrative exposure score	279	0.110	0.054 to 0.167	<0.001*
Perceived narrative credibility score	279	−0.319	−0.368 to −0.271	<0.001*
**Sex assigned at birth**				
Female	279	Reference	—	—
Male		−0.266	−1.461 to 0.930	0.662
Age, years	279	0.048	−0.002 to 0.098	0.061
**Educational attainment**				
Secondary		Reference	—	—
Bachelor's or engineering degree		1.476	0.053 to 2.900	0.042*
Master's degree	279	0.993	−0.215 to 2.202	0.107
Doctoral degree		0.389	−2.736 to 3.514	0.807
Prefer not to answer		2.222	−2.065 to 6.510	0.308
**BMI category**				
<25.0 kg/m²	268	Reference	—	—
25.0 to <30.0 kg/m²		0.980	−0.006 to 1.966	0.051

30.0 to <35.0 kg/m²		1.797	0.401 to 3.192	0.012*
35.0 to <40.0 kg/m²		2.015	−0.207 to 4.237	0.075
≥40.0 kg/m²		3.349	0.910 to 5.788	0.007*
**Daily social media use**	279			
≤1 h/day		Reference	—	—
1–2 h/day		1.083	0.027 to 2.139	0.044*
>2 h/day		0.545	−0.570 to 1.661	0.337

Panel B. Outcome: Attitudes toward obesity pharmacotherapy

**Predictor**	**Model sample size (*n*)**	***B***	**95% CI**	***p*-value**
Narrative exposure score	279	−0.035	−0.101 to 0.030	0.292
Perceived narrative credibility score	279	0.401	0.350 to 0.452	<0.001*
**Sex assigned at birth**	279			
Female		Reference	—	—
Male		0.792	−0.563 to 2.148	0.251
Age, years	279	−0.070	−0.126 to −0.013	0.016*
**Educational attainment**	279			
Secondary		Reference	—	—
Bachelor’s or engineering degree		−1.272	−2.894 to 0.350	0.124
Master’s degree		−1.108	−2.486 to 0.269	0.114
Doctoral degree		−0.956	−4.518 to 2.606	0.598
Prefer not to answer		−0.956	−5.843 to 3.931	0.701
**BMI category**	268			
<25.0 kg/m²		Reference	—	—
25.0 to <30.0 kg/m²		−0.202	−1.329 to 0.926	0.725
30.0 to <35.0 kg/m²		−1.927	−3.523 to −0.330	0.018*
35.0 to <40.0 kg/m²		−1.438	−3.980 to 1.104	0.266
≥40.0 kg/m²		−3.135	−5.925 to −0.345	0.028*
**Daily social media use**	279			
≤1 h/day		Reference	—	—
1–2 h/day		−0.412	−1.619 to 0.796	0.502

>2 h/day		−0.377	−1.652 to 0.899	0.561

Panel C. Outcome: Perceptions of social media communication about obesity treatment

**Predictor**	**Model sample size (*n*)**	***B***	**95% CI**	***p*-value**
Narrative exposure score	279	0.236	0.163 to 0.308	<0.001*
Perceived narrative credibility score	279	−0.009	−0.091 to 0.074	0.835
**Sex assigned at birth**	279			
Female		Reference	—	—
Male		−0.980	−2.581 to 0.621	0.229
Age, years	279	−0.055	−0.123 to 0.012	0.107
**Educational attainment**	279			
Secondary		Reference	—	—
Bachelor’s or engineering degree		−0.374	−2.281 to 1.534	0.700
Master’s degree		−1.277	−2.897 to 0.343	0.122
Doctoral degree		−3.456	−7.644 to 0.733	0.105
Prefer not to answer		−3.289	−9.036 to 2.458	0.261
**BMI category**	268			
<25.0 kg/m²		Reference	—	—
25.0 to <30.0 kg/m²		0.136	−1.225 to 1.497	0.844
30.0 to <35.0 kg/m²		−0.292	−2.218 to 1.635	0.766
35.0 to <40.0 kg/m²		1.498	−1.569 to 4.566	0.337
≥40.0 kg/m²		−0.451	−3.818 to 2.916	0.792
**Daily social media use**	279			
≤1 h/day		Reference	—	—
1–2 h/day		1.174	−0.246 to 2.595	0.105
>2 h/day		0.994	−0.506 to 2.495	0.193

Abbreviations: *B*, unstandardized regression coefficient; BMI, body mass index; CI, confidence interval. Each predictor was examined in a separate univariable linear regression model. Model sample size (*n*) denotes the number of participants with complete data for the outcome and predictor included in the corresponding model. BMI-based models included 268 participants because BMI could not be calculated for 11 participants; all other univariable models included 279 participants. Higher outcome scores indicate, respectively, more favorable views of GLP-1 receptor agonists as an obesity treatment, greater endorsement of negative statements about obesity pharmacotherapy, and stronger agreement with negative appraisals of social media communication. * $p < 0.05$.

Supplementary Table S3. Multivariable linear regression analyses of factors associated with evaluation of GLP-1 receptor agonists as an obesity treatment, attitudes toward obesity pharmacotherapy, and perceptions of social media communication about obesity treatment.

Panel A. Outcome: Evaluation of GLP-1 receptor agonists as an obesity treatment

Model sample size (*n*)	R^2	Adjusted R^2
268	0.465	0.438

Regression coefficients

Predictor	*B*	95% CI	*p*-value
Narrative exposure score	0.072	0.026 to 0.118	0.002*
Perceived narrative credibility score	−0.311	−0.359 to −0.263	<0.001*
Age, years	0.016	−0.026 to 0.058	0.450
**Educational attainment**			
Secondary	Reference	—	—
Bachelor's or engineering degree	0.430	−0.669 to 1.530	0.441
Master's degree	0.170	−0.774 to 1.115	0.723
Doctoral degree	0.588	−1.819 to 2.995	0.631
Prefer not to answer	1.805	−2.154 to 5.763	0.370
**BMI category**			
<25.0 kg/m²	Reference	—	—
25.0 to <30.0 kg/m²	0.664	−0.129 to 1.458	0.100
30.0 to <35.0 kg/m²	1.260	0.174 to 2.345	0.023*
35.0 to <40.0 kg/m²	0.187	−1.586 to 1.960	0.836
≥40.0 kg/m²	1.905	0.001 to 3.809	0.050*
**Daily social media use**			
≤1 h/day	Reference	—	—
1–2 h/day	0.729	−0.101 to 1.560	0.085
>2 h/day	0.438	−0.450 to 1.326	0.332

Panel B. Outcome: Attitudes toward obesity pharmacotherapy

Model sample size (*n*)	R^2	Adjusted R^2
268	0.515	0.492

Regression coefficients

Predictor	*B*	95% CI	*p*-value
Narrative exposure score	0.004	−0.046 to 0.054	0.869
Perceived narrative credibility score	0.401	0.350 to 0.453	<0.001*

**Sex assigned at birth**			
Female	Reference	—	—
Male	0.683	−0.359 to 1.726	0.198
Age, years	−0.041	−0.087 to 0.004	0.074
**Educational attainment**			
Secondary	Reference	—	—
Bachelor’s or engineering degree	0.104	−1.084 to 1.292	0.863
Master’s degree	0.131	−0.894 to 1.156	0.802
Doctoral degree	−0.272	−2.851 to 2.306	0.835
Prefer not to answer	0.445	−3.821 to 4.711	0.838
**BMI category**			
<25.0 kg/m^2	Reference	—	—
25.0 to <30.0 kg/m^2	0.189	−0.672 to 1.050	0.666
30.0 to <35.0 kg/m^2	−1.550	−2.741 to −0.359	0.011*
35.0 to <40.0 kg/m^2	0.397	−1.515 to 2.309	0.683
≥40.0 kg/m^2	−1.375	−3.424 to 0.673	0.187

Panel C. Outcome: Perceptions of social media communication about obesity treatment

Model sample size (*n*)	R^2	Adjusted R^2
279	0.163	0.135

Regression coefficients

Predictor	*B*	95% CI	*p*-value
Narrative exposure score	0.235	0.161 to 0.309	<0.001*
**Sex assigned at birth**			
Female	Reference	—	—
Male	−0.614	−2.153 to 0.926	0.433
Age, years	−0.072	−0.139 to −0.006	0.033*
**Educational attainment**			
Secondary	Reference	—	—
Bachelor’s or engineering degree	−0.682	−2.473 to 1.108	0.454
Master’s degree	−1.129	−2.656 to 0.399	0.147
Doctoral degree	−2.307	−6.256 to 1.642	0.251

Prefer not to answer	−2.935	−8.351 to 2.481	0.287
**Daily social media use**			
≤1 h/day	Reference	—	—
1–2 h/day	0.664	−0.681 to 2.009	0.332
>2 h/day	0.502	−0.934 to 1.937	0.492

Abbreviations: *B*, unstandardized regression coefficient; BMI, body mass index; CI, confidence interval; R^2, coefficient of determination. Model sample size (*n*) denotes the number of complete observations included in the corresponding multivariable model. Higher outcome scores indicate, respectively, more favorable views of GLP-1 receptor agonists as an obesity treatment, greater endorsement of negative statements about obesity pharmacotherapy, and stronger agreement with negative appraisals of social media communication. Model diagnostics provided no evidence of multicollinearity (all variance inflation factors <5), departure from normality of the residuals (all Shapiro–Wilk test *p*-values >0.05), or heteroscedasticity (all Breusch–Pagan test *p*-values >0.05). * $p < 0.05$ based on unrounded *p*-values.
